# A Realist Evaluation of Social Care Practitioners’ Experiences With and Understanding of Applied Healthcare Research

**DOI:** 10.1177/00469580241248130

**Published:** 2024-05-24

**Authors:** Gurkiran K. Birdi, Geoffrey Wong, Rachel Upthegrove, Suzanne Higgs, Annabel Walsh, Amy Ahern, Katherine Allen, Jo Howe, Hafsah Habib, Karen Nixon, Sheri Oduola, Ian Maidment

**Affiliations:** 1 Aston University, Birmingham, UK; 2 University of Oxford, Oxford, UK; 3 University of Birmingham, Birmingham, UK; 4 The McPin Foundation, London, UK; 5 University of Cambridge School of Clinical Medicine, Cambridge, UK; 6 Birmingham and Solihull Mental Health Foundation Trust, Birmingham, UK; 7 Midlands Partnership NHS Foundation Trust, Stafford, UK; 8 University of East Anglia, Norwich, UK

**Keywords:** social care, evidence-based practice, realist evaluation, severe mental illness

## Abstract

Social care practitioners are often under-represented in research activity and output. Evidence-based practice enables social care practitioners to develop/engage the skills to evaluate evidence and be more actively involved in research. REalist Synthesis Of non-pharmacologicaL interVEntions for antipsychotic-induced weight gain (RESOLVE) is a NIHR-funded study where realist synthesis is used to understand and explain how, why, for whom, and in what contexts non-pharmacological interventions help service users, with severe mental illness, to manage antipsychotic-induced weight gain. Social care practitioners are a key part of the team providing care for people living with severe mental illness and therefore supporting antipsychotic-induced weight gain. The current study, RESOLVE 2, uses realist evaluation and RESOLVE as an illustrative example to help understand why and how social care practitioners engage (or not) with research. Semi-structured, audio-recorded interviews will be undertaken with a purposive sample of approximately 20 social care practitioners working with people who have severe mental illness, are treated with antipsychotics, and have experienced weight gain. Participants will be recruited from NHS Trusts and recruitment avenues such as social media and personal networks. Topics discussed during interviews will include barriers and facilitators to engagement in research, current, and past engagement as well as recommendations for researchers and other practitioners. Interview recordings will be transcribed verbatim and analyzed using realist evaluation which will allow in-depth causal explanations for research engagement. Better understanding of research engagement by social care practitioners will allow for evidence-based practice and better patient outcomes within these settings.

**What do we already know about this topic?**
Social care practitioners have low levels of engagement with research as well as perceived low confidence levels in the application of research skills/knowledge to their role.
**How does your research contribute to the field?**
This study uses realist evaluation—an approach not used before to explore this topic—to understand why and how social care practitioners engage with research activity.
**What are your research’s implications toward theory, practice, or policy?**
This qualitative study will inform recommendations made to SCPs, policy makers, including national policy makers and funding organizations on how we can build research engagement in the social care sector.

## Introduction

This qualitative study aligns with an ongoing NIHR-funded study, RESOLVE^1^ which uses realist synthesis, combining primary and secondary data collection to understand and explain how, why, for whom, and in what contexts non-pharmacological interventions help service users to manage antipsychotic-induced weight gain. Preliminary findings from RESOLVE suggest the importance of working across organizational sectors, such as social care, to support those with antipsychotic induced weight gain. RESOLVE 2, the current study, focuses on understanding the engagement (or not) of SCPs in research and findings will be used to develop and promote uptake of the RESOLVE guidance; RESOLVE 2 will entail a realist evaluation using qualitative semi-structured interviews with SCPs; it will use RESOLVE as an illustrative example.

The National Institute for Health and Care Research (NIHR) defines social care practitioners (SCPs) as those working in social care or social work^2^ (eg, social workers, occupational therapists, care workers, supervisors, and managers). SCPs are often key members of the team providing care for people living with SMI and are frequently involved in supporting these people through their treatment and recovery.^3^ SCPs are often involved in the co-production of recovery pathways and complex interventions designed to address the weight gain and metabolic dysfunction that is often seen in those with SMI.^4^ These interventions have been found to produce positive effects such as improved quality of life and weight management in those with antipsychotic-related weight gain.^5^ Nonetheless, SCPs are often not aware of the evidence that informs these interventions and recovery pathways.^6^

In health and social care settings, evidence-based practice (EBP) describes making decisions about care based on the best, currently available scientific evidence.^7^ EBP allows for better quality of care, better outcomes for patients and service users and improved overall practitioner performance.^8,9^ Within healthcare settings including primary and secondary care, EBP is applied to a much greater extent in comparison to social care; this in turn results in less scholarly and research activity from SCPs.^10-12^

Current literature focusing on SCPs has found low levels of engagement with research as well as perceived low confidence levels in the application of research skills/knowledge in their social work.^13,14^ Recently, the NHS has prioritized social care research to address this imbalance and initiated a specialist NIHR School for Social Care Research^14,15^ It is imperative for SCPs to be equipped with the necessary skills and knowledge to access, interpret, assess, synthesize, and evaluate evidence, as well as have opportunity to engage with research activity.^13,16^ Studies carried out in other countries such as Australia and USA have indeed found similar themes; allied health professionals engage in research to a lesser degree compared to medical practitioners.^17^ Reasons cited in this literature include lack of organizational support,^18^ lack of research knowledge and training^19^ in addition to available opportunities for engagement in research.^20^

RESOLVE 2 is focused on research engagement by and with SCPs to build impact across the social care sector. Using RESOLVE as an illustrative example, we want to understand how researchers can ensure that relevant healthcare research is taken up and utilized by SCPs, underpinned by pro-active dissemination. As highlighted previously, research can help health and social care practitioners, and policy makers improve the services they offer. This research project attempts to understand and address the research engagement gap amongst SCPs, focusing on those working with people living with severe mental illness (SMI). From this we will produce guidance for researchers and SCPs and use findings to develop and promote uptake of the RESOLVE guidance. In RESOLVE 2, realist evaluation will be used as this is a theory driven approach to explain how real-world interventions operate in complex social systems, linking outcomes to contexts and underlying causal processes that is, mechanisms.^21^

### Aim of the Study

Generate theory-based explanations to help better understand how SCPs working with people living with SMI engage in research by using RESOLVE as an illustrative example. We will then use these findings to develop recommendations on how research uptake can be facilitated in SCPs.

## Methods

### Study Design

This realist evaluation will collect data from realist interviews conducted with SCPs who work with people living with SMI and have experienced weight gain following antipsychotic medication.

Realist evaluation has been used increasingly in health services research to explore and evaluate complex health system interventions.^22^ The interviews will be semi-structured; an interview guide will be developed. The RAMESES II (Realist and Meta-narrative Evidence Syntheses: Evolving Standards) reporting guidelines will be used to structure the reporting of the study methods and data analysis.^22^ Underpinned by the principle that context (C) will trigger mechanisms (M) to cause outcomes (O), a realist evaluation goes beyond focusing purely on inputs and outputs. It involves exploring and identifying the mechanisms (ie, causal processes) that links inputs to outputs and recognizes the need for particular conditions (or contexts) to be present for the causal mechanisms to be triggered and cause a particular outcome. The relationship between context, mechanism, and outcome is presented as a “CMO configuration.”^21^ Based on these principles, we want to identify CMO configurations that will explain how, why, and to what extent SCPs engage with research using RESOLVE as an illustrative example.

The study will also be supported by a Stakeholder Group (SG) comprising SCPs and appropriate study team members. The SG will help shape the trajectory of the research and assist with the development and refinement of interview questions. A key role for the SG will be to help us to refine our dissemination strategy and to support the pathway to impact. The SG will also review our recommendations for engaging SCPs in research.

### Eligibility Criteria

#### Inclusion criteria

UK-based SCPs [as defined by the NIHR as anyone working/having worked within social care or social work^2^; (NIHR, 2023)] supporting people with SMI currently taking or previously taken antipsychotics with experience of antipsychotic induced weight gain. Severe mental illness (SMI) in this study includes people with psychological problems that are often so debilitating that their ability to engage in functional and occupational activities is severely impaired.^23^ Some examples of SMI are schizophrenia, schizoaffective disorder, bipolar disorder, and all other non-organic psychoses. SCPs are eligible to take part in the study if they have experience of supporting those with SMI, regardless of duration of SMI, age, medication, and other factors.

#### Exclusion criteria

Health care professionals (eg, doctors, pharmacists, and nurses).

SCPs who have no experience of supporting people with SMI currently taking or previously taken antipsychotics who have experienced weight gain associated with antipsychotics.

Participants who do not work as SCPs in the UK.

### Sampling

A purposive sampling approach will be used where participants will be purposively sampled to ensure diversity in potentially conceptually relevant characteristics including, for example, locality (rural vs. urban), and the index of deprivation in the area that they work, gender, and ethnicity. This will allow us to capture the voices of different people to explore “what works for whom”; a component integral to realist evaluation.^22^

### Size of Sample

The predicted sample size, informed by RESOLVE and previous experience, is around 20 SCPs, however we will continue interviewing until no new theoretical insights to the broad topics in the interview guide are identified in the interviews. As the interviews are to be conducted by 1 researcher, the researcher will note the point at which there is an absence of new themes or concepts.

### Study Setting

The interviews will be conducted either face-to-face in a convenient location for participants, or if required, using videoconferencing (eg, MS Teams) or via telephone.

### Recruitment

Potential participants will be invited to contact the researcher for study information and to confirm eligibility. Specific recruitment strategies to identify potential participants include NHS Trusts acting as Participant Identification Centers (PICs); within these Trusts, study information will be promoted via direct email to eligible staff. Currently, the agreed Trusts acting as PICs will be Birmingham and Solihull Mental Health Foundation Trust (BSMHFT) and Midlands Partnership NHS Foundation Trust (MPFT). We will also advertise the study on social media platforms such as Twitter, use our professional networks to identify participants and advertise through independent social care providers and local authorities. Emails advertising the study will be sent to these third-sector organizations to identify eligible participants. Participants will be sent consent forms and participant information sheets via email, after which they will be able to arrange a suitable time and place for the interview to take place. Participants will be reimbursed with £30 shopping vouchers for their time in the study. As recruitment of participants is often unpredictable, other avenues may be sought, such as snowballing techniques, SCP social networking platforms, and other NHS Trusts may be sought as act as PICs for the study. Data collection using semi-structured interviews is ongoing and due to complete in early-mid 2024.

### Data Collection

The interview guide to be developed will be based on the aims of the study and concepts identified in existing questionnaires and guides from reviewing the literature.^13,14^ The main topics have been summarized in Table 1. Semi-structured realist interviews will involve starting with general questions about the interviewee’s role, experiences, and views about the theory. Subsequent questions will be guided by their responses. Specific elements of the theory will be introduced and tested with the participant. As interviews progress and knowledge of theory grows, the questions may evolve and become less standardized and focus on using questions to collect the data needed to refine specific CMO configurations.^23^ The topics in the interview are not sensitive and are not expected to be a cause of any distress or upset in participants. This study has received a favorable ethical opinion from an NHS Research Ethics Committee and has Health Research Authority approval. Participants will provide informed electronic consent prior to the interview and will be sent participant information sheets via email.

**Table 1.** Summary of Topics Covered in Interviews.

Previous and current engagement in research
Importance of research activity to SCPs
Barriers and facilitators to engagement and uptake of research using RESOLVE as an exemplar.
Recommendations for researchers and other SCPs

### Data Analysis

Realist evaluation data will be analyzed using NVivo, a qualitative analysis software that allows researchers to manage, analyze, and visualize qualitative data such as interviews systematically and individually. Manzano^23^ notion of theory gleaning, refinement and consolidation will be applied during the interview process and analysis. Initial literature scoping and meetings with the research team and SG will help identify some preliminary program theories (stage 1). These initial program theories will be tested (confirmed, refuted, and refined) against the interview data and help develop some “if-then” statements. We will work collaboratively in the research team to code and analyze interview data; calibration exercises will be conducted to ensure consistency and inter-coder reliability. Two researchers in the team will independently code a subset of data and compare results to identify discrepancies and establish consensus. The research team will also hold regular meetings to discuss coding progress, share insights, and address any challenges or uncertainties. By following these steps and incorporating principles of transparency, collaboration, and reflexivity, coding between researchers, we can contribute to the generation of robust, credible, and contextually sensitive findings.

Prior to the coding on NVivo, transcripts will be read to gain a better contextual understanding of the interviews. Each interview will be treated as an individual data source and within each source “nodes” will be created to capture data that may inform potential CMOs. Initial coding will involve identifying patterns, themes, and recurring concepts in the interviews. We will look for patterns related to the contexts in which SCPs engage with research, the mechanisms that facilitate or hinder engagement, and the outcomes of engagement^22^; this will be done continually and will determine the topics discussed in forthcoming interviews. Data will then be categorized according to the context, mechanism triggered by the context, and outcome produced. This step involves looking for patterns that explain why and how SCPs engage with and understand research. Then, as coding progresses, we will refine and revise the C-M-O configurations by examining how different contexts (eg, time constraints) interact with mechanisms (eg, feelings of frustration) to produce specific outcomes. Throughout the coding and analysis process, we will engage in continuous reflection and iteration, revisiting, and refining earlier stages based on new insights and emerging evidence. In our findings section, we will present these CMOs alongside verbatim quotes to substantiate our interpretations. The table below illustrates the steps that will be taken throughout the analytic process (Table 2):

**Table 2.** Steps Undertaken for Realist Evaluation.

Step	Description
1. Initial program theory	Develop an initial program theory that outlines the underlying mechanisms, contexts, and outcomes for SCPs’ engagement with research^21^
2. Data collection	Collect data through semi-structured interviews with SCPs^22^
3. Initial data analysis	Analyze the collected data using qualitative approaches to identify patterns, themes, and recurring concepts^22^
4. Context-mechanism-outcome (CMO) configurations	Identify Context-Mechanism-Outcome (CMO) configurations that explain how the research understanding and engagement in different contexts by triggering mechanisms to produce outcomes.
5. Refine program theory	Refine the initial program theory based on the emerging CMO configurations
6. Triangulation	Triangulate findings by comparing data from multiple sources and methods to validate emerging patterns and CMO configurations.
7. Testing and validation	Test the refined program theory through further data collection and analysis to validate its predictive power across different contexts.
8. Continuous iteration and reflection	Engage in continuous iteration and reflection throughout the evaluation process, refining the program theory based on new evidence and insights.
9. Dissemination and utilization	Disseminate the findings of the realist evaluation to stakeholders and use the insights gained to inform decision-making, practice, and future interventions.

## Discussion

The experiences of SCPs with research are varied and complex, reflecting the multifaceted nature of the social care profession itself. Through the exploration of their encounters with research, we expect several key program theories to emerge. As mentioned above, SCPs often lack the opportunity, capacity, and skill set capability to access and apply evidence and only a few go on to undertake research.^24-26^ Using the methodologically rigorous realist evaluation, RESOLVE 2 will generate theory-based explanations to understand why the lack of research engagement and understanding exists in social care. Previous studies have tended to use questionnaires with fixed-response questions or qualitative methods such as thematic analysis which do not allow for in-depth causal explanations.^14,27-29^ This realist evaluation will yield mechanistic insights into the individual contexts and specific barriers and facilitators experienced by SCPs in relation to engaging with research. Whilst there are limitations to only using qualitative data from realist interviews to understand SCP engagement with research, a strength of our approach is the in-depth understanding we can gather, especially when the lack voices from social care professionals in the academic literature are factored in. This knowledge can then be shared with our SG to co-produce guidelines to enhance SCP engagement with research. Using this methodology will also complement realist approaches used in RESOLVE which is used as an illustrative example in this study. Qualitative data collection methods offer rich insights into individuals’ experiences, perceptions, and behaviors. However, they also come with several limitations such as replicability issues,^30^ subjectivity, and bias.^31^ It is also important to highlight some limitations of using realist evaluation in particular; it produces context-specific findings which can limit the generalizability of findings across different settings.^21^ Within realist evaluation, identifying, and measuring mechanisms, particularly latent or underlying processes, can be challenging, which may affect the validity and reliability of findings.^23^

As we will recruit SCPs working both within and outside the NHS, we will interview participants from diverse backgrounds, occupations, and experiences. We will do so by targeting recruitment to diverse and hard to reach communities by using a range of recruitment strategies. We also anticipate that the findings of this study and subsequent guidelines will help researchers in the field acknowledge and address the discrepancies between SCP high levels of interest and low levels of engagement in research.^14^ Although we will only recruit SCPs based in the UK, the findings may have some relevance in other countries with similar healthcare systems.^17^

In conclusion, the experiences of social care practitioners with research underscore the importance of bridging the gap between academia and practice in the field of social care. By exploring current knowledge and engagement, we can produce guidance to enhance research-informed culture that improves the quality of social care services and ultimately outcomes for vulnerable individuals and communities.
